# Retrieving memories of dialogical knowledge production: COVID-19 and the global (re) awakening to systemic racism

**DOI:** 10.1177/1350506820951937

**Published:** 2020-11

**Authors:** Chantelle Lewis

**Affiliations:** Department of Sociology, Goldsmiths, UK

The origins of my approach to academic scholarship were established by my parents’ commitment to the revolutionary politics of social change and care. Though their perspectives differed in terms of their individual and structural analyses – whether through popular culture, the news, or reflections about the persistence of inequalities – dialogical interventions about the *possibility* of a more equitable society were instilled into my consciousness from a young age.

I grew up in a predominantly white suburban area in the UK (West Midlands), where my parents’ emancipatory discussions were not just obsolete, but both monitored and condemned by the adults and young people around me. I observed the 1990s and early 2000s through the cynicism of many of these people; a cynicism no doubt born of the variety of lived socio-economic disparities many (including my own family) endured. With these pertinent, but ongoing, observations, I always found it difficult to see what my position might be within collective efforts to change the oppressive structures I witnessed and was disproportionately exposed to.

I was not a ‘high-achieving’ young person; I found reading and writing exceptionally challenging throughout school and university (I still do!). I always struggled to understand how I would be taken seriously if I failed to ‘achieve’ within the limited scope of the UK education system. Later in my adult life, I found out that I have a number of neurodiverse traits (high functioning ADHD, dyspraxia and dyslexia), which helped to explain why I regularly felt that I lacked the intellectual capacity to adequately contribute to real social change. Growing up with limited social, cultural and economic capital, I found myself consistently positioned on the periphery of academic achievement (reading and writing), but my oral ability has remained a relative strength (that I attribute to my high functioning ADHD). My good friends (and co-hosts and co-founders of the Surviving Society podcast) – Tissot Regis and George Ofori-Addo – and I often talk about the power of discussion. In our ongoing quest to convince publics that our methods of engagement are legitimate, we often discuss the need to *speak* to people; we need the space to *vocally* persuade.

You may be asking yourself why this brief and seemingly self-indulgent overview of my life is relevant to reflections on hopeful possibilities during and beyond the global COVID-19 pandemic. I am informed by the scholarly interventions by Black feminists like Patricia Hill Collins who encourages a reflection on how *dialogical knowledge production* (often connecting theory, practice and action) creates memories of praxis that can be relied on in times of crisis, but also during moments of imagining alternative futures (Hill Collins, 2012). As she notes:

My work encapsulates multiple situated standpoints – distinctive, competing, and often contradictory angles of vision that shift not only when I vary physical and intellectual social locations but also when times change around me. While it has been tempting to simplify my situated standpoints and merge them into a homogeneous narrative to make the world more comfortable for me, my challenge has been to sustain a commitment to dialogical knowledge production, especially in situations of conflict. (Hill Collins, 2012: 14–15)

This quote from Hill Collins summarises how I feel about dialogical knowledge production; it encompasses a journey with no distinct destination. I have continuously sought to find growth whilst being challenged by the knowledge production I have engaged with through reading, writing, talking, and listening. At times, I find it difficult to evade the homogeneous narratives Hill Collins refers to, but this is part of the journey. For me, a commitment to dialogical knowledge production means finding comfort in the *unknowing* of social life; these are the moments where you are challenged to think differently, more critically and more carefully.

The combination of neurodiversity and an over-exposure to the racialised and classed structures of UK academia (and education more broadly) has meant my journey into understanding the possibilities of dialogical knowledge production has been repeatedly delayed and hindered. My attempts to harness the benefits of formal education as an emancipatory tool have been consistently redirected by what my friends Paulette Williams and Jason Arday and I recall as *through the backdoor*; as opposed to the more convenient routes where one can walk through the front doors of formal educational institutions (and perhaps even take a seat in the reception area before being guided through the house!).

This notion of *the backdoor* is something with which many Black and people of colour from working-class backgrounds will be all too familiar. It is extremely difficult to adequately describe what this process is like without it seeming like we have been ‘lacking’ in some way. Many of us were raised amongst kin in cultures that are enriching, challenging and liberating. We exist within populations of resilience since so many of our ways of being have been persistently rejected by those with power. Although I wish to strongly advocate for Black people to be able to lead ordinary and restful existence*s*, right now, in these moments of extreme uncertainty, I am feeling particularly grateful that I have had to learn how to make my own way *through the backdoor*.

At the time of writing this, the world continues to be in the midst of the COVID-19 global pandemic, and we have just passed four weeks of the renewed Black Lives Matter movement protests and demonstrations, triggered by the murders of Breonna Taylor, George Floyd and Ahmaud Arbery at the hands of police in the United States of America. Within the UK context, many have been protesting, advocating and (continuously) expressing the need to examine and eradicate the violence of white supremacy. During this time, we have witnessed a corporate and institutional recognition of Black Lives Matter(ing), and in many cases, what feels like a reactionary response from liberal organisations on what they can do to tackle their own contributions to the racialised subjugation of Black people. Many of us who have been fighting for equity and the destruction of white supremacy prior to this moment have been relentlessly contacted as ‘experts’, positioned to facilitate and support people’s lifelong commitment to post-racialism (Bonilla-Silva, 2010; Goldberg, 2013; Lentin, 2014; Valluvan, 2016). We have had to witness those at the forefront of racialised capitalism – who economically and ecologically profit from the destruction of Black life – claim that they do care about global racial subjugation . . . after all. Now, many critics might be reading this and thinking (or shouting at the screen!) ‘Well what do you want us to do? We are listening *now* . . .’. I do not have a straightforward answer, but I would contend that there is a distinctive symbolic violence caused by a white awakening to systemic racism. For many (myself included) this process has been exhausting, confusing, scary, and hopeful all at the same time. I am learning to reject the approval of systems of white supremacy, but I do strive for freedom for as many of us as possible – if those with power are waking up, does that mean fewer people will suffer? Whilst we navigate the ongoing impact of the global COVID-19 pandemic and more people continue to discuss whiteness, racism and Black lives, many of us seeking global emancipatory futures are picking up the maps we used to find our way *through the backdoor*. These maps help us survive, but they also enable us to actively invest in dialogical knowledge production in times of crisis.

I regularly remind myself (or am reminded by friends) to recognise that revolutionary ideas and practices need a plethora of roles and perspectives. This is not an argument for a ‘marketplace of ideas’, but a recognition that freedom for the structurally oppressed requires multidimensional theorisations, conceptualisations and practices. Appreciating the multi-layered conditions needed for social change has been an ongoing and emotional journey for me. Immersing myself in bell hooks’ scholarship, and listening to and reading Dr Lisa Palmer’s (2018) personal and political conceptualisations of love, community and resistance are just some of the ways I’ve been able to comprehend complex social processes of acceptance, empathy and understanding (hooks, 1999, 2001, 2002; Palmer, 2018, 2020).

This is where I think I can be of use in this revolutionary moment and the ones yet to come. I am striving to be among the many voices who remind people that those of us on a quest for social change are part of a collective of varying modes of operating. The tools to reconcile differences within our broad coalition require a combination of accountability, reflectiveness and reflexivity. Dialogical knowledge production through conversation, reading and action are what will continue to help us all make sense of how we make the best of and understand our differing roles.

Just one month before the UK lockdown was imposed by the government in response to the COVID-19 pandemic, I met with Dr Aviah Sarah Day – scholar-activist and part of Sisters Uncut. Sisters Uncut are a group of women and gender-variant people who use direct action to fight domestic sexual, gendered and state violence in the UK. We were recording a podcast as part of the ‘Surviving Society Alternative to Woman’s Hour’ series (Lewis and Day, 2020). I look back fondly on our conversation about Aviah’s activism, as a feminist memory of critique (Ahmed, 2017). It was one of those moments in which I needed to be reminded of the varying roles required for substantive social change. Our talk focused on how Aviah had embedded both her personal and professional life into her work to end abuse against womxn and create alternative visions for a society grounded in the politics of abolition (Day and Gill, 2020). Before our conversation, I was still mourning the UK General Election result of December 2019, when the Conservative Party won a landslide majority. I felt like we (Labour Party organisers who were invigorated by the possibilities of socialism) completely lost the decade-long battle against the state’s relentless neglect and mistreatment of working-class populations. At the start of 2020, I felt physically and emotionally drained by the electorate’s rejection of the possibility of a more equitable society.

Through dialogical interventions about recovering from this renewed sense of loss, Aviah helped me return to a key role that I can play. It is a role that I do not believe everyone should have to negotiate, particularly members of oppressed and marginalised groups – because it requires an active appreciation that many of the people who voted *against* equity *for all* will still be needed in the fight for emancipatory freedoms. Of course, our reflections during the podcast were grounded in the theory and action of the many revolutionaries who have fought for social change before us – we stand on their shoulders. But I needed this conversation *in that moment*.

I am sure many people can recall similar life-changing conversations with people they admire and respect. Conversations that drive you to act and challenge you to think harder and more critically about your position(s) in the world. Little did I know that this conversation would quickly become one of the *memories of dialogical knowledge production* that would allow me to better understand the role I would play during the COVID-19 global pandemic and the 2020 version of the global awakening to systemic racism.

The unexpected events of 2020 (so far) have necessitated an incorporation of many of the dialogical interventions that have shaped my perspective(s) into my ‘daily survival toolkit’. I have sought to channel my anger about how materially and socially uneven this moment is into varying modes of activism. I am guided by Audre Lorde’s ‘The Uses of Anger’ (1981; see Lorde, 1997) and more recently, Brittany Cooper’s conceptualisation *Eloquent Rage* (2018). In the current UK context, I am also inspired by Kelechi Okafor’s poignant observation of how anger can be used ‘to channel the personal and collective politics of hope’ (Okafor, 2020).

In the UK context, an ongoing awareness of pre-existing social and economic disparities deepened through decades of inhumane social policies – particularly those felt by Black and Brown working-class communities – should inform future dialogical knowledge production during this global crisis (Emejulu and Bassel, 2018; Tyler, 2020).

Recognising the inequitable pressures and outcomes (for life and death) that COVID-19 brings to the fore for marginalised populations has necessitated a return to recent dialogical interventions about the social and material contexts that pre-date this moment. In relation to the UK, I have been inspired by the scholars who have tirelessly written about and campaigned to address the raced and classed dimensions of Brexit (Benson, 2019; Bhambra, 2017; Dorling, 2016), the disproportionate impact of austerity on Black and Brown women (Emejulu and Bassel, 2018; Gayle, 2020; White, 2020), and the reinvigoration of nationalist sentiments (Begum, 2020; Emejulu, 2016; Virdee and McGeever, 2018), especially those afflicting the lives of Black and Brown Muslim womxn in the UK (Johnson et al., 2018). These unevenly felt racialised and classed disparities should inform how we understand the urgencies of the present moment. Understanding the multi-layered approach and praxis of real social change requires underlining the fact that we have not all arrived into the present with the same experiences and material conditions, even if we believe ourselves to be true allies during the revolution. We must ask ourselves and reflect on our differentiated starting points.

Lola Olufemi’s voice and writing remind me of the multiple ways that equitable freedoms are grounded in ongoing learning and imaginative processes (Olufemi, 2020). In particular, her work has prompted me to reflect on the many collectives that publicly and privately tend to agree on the violence of capitalism, white supremacy and heteronormative patriarchal conditions, but follow different routes when fighting and advocating for their destruction. Together with bell hooks’ *Love Triology*, I reckon with Olufemi’s imagining whilst reminding myself (and those around me) that some of us will never be at peace with the fact that there will be variances within our approaches, and this is something we must accept, but also build on and learn from. However, this is not about being an apologist for (or accepting) hateful and harmful discourses (and movements) that might present within these broad coalitions. For example, we are witnessing a renewed global threat to trans, non-binary and gender fluid populations (especially here in the UK), so if you have become apathetic, or are ignoring these forms of violence, you have underestimated how integral they are to upholding structures of unequal power as we imagine and build towards the conditions for our *collective* emancipation.

As I did during my formative years, listening to my parents’ anticipatory narratives about the possibilities of radical politics, I feel privileged that many of my conversations with people around me now focus on what our different roles might be to achieve a more just and equitable society. As we constantly ask ourselves and each other: What could our ever-evolving approach to freedom for structurally marginalised populations look like? It is in these moments that I always return to the memories of dialogical interventions that inform, challenge and critique my assessments of the systems needed for more equitable societies.

We need hope to survive this moment, which is why I end these reflections with a list of some local UK activist and scholarly interventions that have connected theory and practice. They represent just a handful of organisations committed to equitable futures that have been actively responding to the uneven impact(s) of COVID-19: The Ubele Initiative, Morecambe Bay Poverty Truth Commission, The Monitoring Group, Sistah Space, MAIA group, Racial Justice Network and Kids of Colour.

Our dialogical interventions beyond this moment do and should continue to incorporate the varying ways in which radical scholars, activists, artists and community organisations continue to respond to the classed and racialised emergencies that the pandemic presents and exacerbates.
